# Evaluation of a New Home Patient Services Practicum for Community Pharmacy Students in Japan

**DOI:** 10.3390/pharmacy9030120

**Published:** 2021-06-29

**Authors:** Rie Kubota, Junichi Mukai, Michiko Yamada, Yurika Yoshino, Nakaba Okamura

**Affiliations:** Clinical Pharmacy Education, Research and Education Center for Clinical Pharmacy, School of Pharmacy, Kitasato University, 5-9-1 Shirokane, Minato-ku, Tokyo 108-8641, Japan; mukai11@kitasato-u.ac.jp (J.M.); m-shrht@kitasato-u.ac.jp (M.Y.); yoshinoy@pharm.kitasato-u.ac.jp (Y.Y.); okamuran@pharm.kitasato-u.ac.jp (N.O.)

**Keywords:** qualitative analysis, home patient service, clinical pharmacy education, pre-practical training

## Abstract

This study aimed to evaluate the usefulness of the practicum as well as assess the knowledge, skills, and various specific realizations that the students gained from it. A total of 244 students role-played a scenario in which a pharmacist visited a patient at home and provided pharmaceutical management services. After completing the practicum, the students completed (i) a questionnaire survey consisting of six questions that assessed their level of understanding of the role of pharmacists in home medical care and (ii) a rubric survey that evaluated their learning achievement. In addition, they submitted practicum portfolios describing the patients’ living conditions, physical conditions, and background as well as the services that required consideration of said variables. Their responses to the portfolio item “What were noticed through the practicum” were analyzed using the grounded theory approach. After the practicum, 45% and 53% of the students reported having a full and partial understanding of a pharmacists’ role in home medical care. The students’ mean ± standard deviation rubric score was 3.0 ± 0.4. They classified monitoring drug use, support for improving medication adherence, and observation to identify side effects early as major service categories in home medical care. The practicum led the students to perceive the need for communication with patients and various healthcare professionals to improve their readiness for practical training.

## 1. Introduction

Individuals aged 65 years and older account for 28.4% of the population of Japan’s aging society [1]. Consequently, the focus of medical needs has changed from acute illnesses to chronic diseases, including cancer, and the need to remodel the medical care system from the conventional hospital-centered system to an integrated community care system, in which medical care, nursing care, preventive care, housing, and lifestyle support services are provided in a coordinated manner, has become urgent [2]. Home medical care is a system in which healthcare professionals conduct home visits to patients who cannot visit a hospital/clinic and provide them with holistic and comprehensive medical care, and its importance is increasing. In home medical care, medical professionals, such as medical doctors, pharmacists, and nurses, as well as professional caregivers work together in a multidisciplinary, collaborative manner to treat and care for patients. The Japan Pharmaceutical Association has stated that pharmacists should not only supply medicines but also provide other services to optimize the efficacy and safety of drug therapy for home care patients, including modifications in dispensing manners depending on a patient’s condition (e.g., dispensing drugs in single-dose packages); management of medication history (e.g., checkups for unused medicines and drug interactions); instruction/assistance on medication; monitoring of adverse reactions; and making prescription proposals to the responsible medical doctors [3].

In Japan, in addition to the public health insurance system, a public long-term care insurance system to which all individuals in the country are subscribed is in place. Medical service fees for home medical care are reimbursed from either of the two systems, and these systems do not differ in terms of the specific pharmaceutical management and consultation services provided by pharmacists. For patients who require both medical care and long-term care, the use of the long-term care insurance system for the reimbursement of medical fees is preferable to that of the health insurance system. Of the approximately 59,000 pharmacies in Japan (as of 2016), 11% and 33% provide services for home care patients under the health insurance and long-term care insurance systems, respectively [3]. Furthermore, the total number of home visits for drug management and consultation services provided by pharmacies increased to 7.91 million in 2016 [3], highlighting the increasing and developing role of pharmacists in home medical care [4,5,6]. In fact, inadequate storage of drugs, medication nonadherence (missed doses), and the lack of understanding of prescribed medicines were reported by 46.1%, 40.5%, and 36.5%, respectively, of patients undergoing medical treatment at home [7]. Various drug-related problems, such as the use of multiple medications (polypharmacy), are particularly more common in geriatric patients [8]. In other countries, pharmacists would typically visit care or nursing homes but would rarely visit patient homes and provide home medical care [9]. In the United States, pharmacist-led home visits are currently not a standard practice in the community pharmacy setting [10].

The current 6-year pharmacy education system in Japan was launched in 2006 [11], and all universities provide education according to the model core curriculum [12]. Fourth-year students undergo pre-practical training involving simulation training on the various duties of pharmacists, followed by computer-based testing and an objective structured clinical examination (OSCE) that includes practical tests on six tasks in five categories [13], to assess their preparedness for practical training in terms of knowledge, skills, and attitudes. The “pharmaceutical management at home” task was added to OSCEs in the 2018 fiscal year. Students who pass both tests can enroll in hospital and pharmacy practicums lasting 11 weeks each in their fifth year of study.

The general instructional objective of the fifth-year practical training in pharmacies is for students to practice drug therapy as well as acquire the basic skills necessary to participate in team medical care or community health and medical care, which would prepare them for an active role as a pharmacist in the clinical setting from the perspective of patients/recipients. Participation in local health and medical care as well as welfare services, including home medical and nursing care, and learning about team medical care in the community setting are integral elements of students’ practical training. Therefore, the Kitasato University School of Pharmacy (Tokyo, Japan) recently incorporated a practicum in home patient services in the fourth-year pre-practical training course.

The objectives of this study were to evaluate the usefulness of the practicum and to determine the knowledge, skills, and various specific realizations that the students gained during the program. We analyzed their responses to a questionnaire survey and a rubric survey after they completed the practicum. In particular, we qualitatively analyzed their practicum portfolios with regard to their personal realizations about their practicum experience.

## 2. Materials and Methods

### 2.1. Participants

The inclusion criteria were fourth-year students enrolled at the Kitasato University School of Pharmacy who had completed the home patient services practicum. A total of 244 students participated in this study. Among them, 63 were male, and 181 were female. The exclusion criteria were as follows:

1. Those who did not give consent to the questionnaire survey
2. Those who did not allow use of their submitted portfolio for research

Since this study was a survey using questionnaire surveys and portfolio analyses, we did not obtain written or oral consent from the study subjects. However, subjects have been given the opportunity to refuse to participate in the research in accordance with the “Matters to be disclosed regarding the implementation of the research when they do not receive informed consent” indicated in the ethical guidelines for medical research targeting humans. Therefore, we have posted materials on opt-out. We have administrated this survey for one year. This study was approved by the Kitasato University Research Ethics Committee (protocol code 18065; data of approval, 1 October 2019).

### 2.2. Overview of the Practicum

The fourth-year students underwent 14 practicum courses for 8 months as their pre-practical training in various duties of pharmacists, including drug information analysis, physical assessment, drug dispensing, intravenous drug preparation, patient counseling in hospital and community pharmacies, as well as home patient services. The students were divided into small groups and rotated through the practicum courses. Their knowledge was assessed using written examinations, and their skills and attitudes were evaluated by faculty members at each practicum. After completing each practicum course, the students were asked to evaluate themselves using a rubric and to submit a portfolio for assessment.

For the home patient services practicum, the students role-played a scenario in which a pharmacist visited a patient at home and provided pharmaceutical management services. A corner was set up in the laboratory room such that it simulated the home setting, with the patients sitting in wheelchairs. The students, as pharmacists, entered the room, sat in front of the patient, and conducted an interview and observation study.

The goal of the practicum was to develop the students’ capability to use appropriate procedures in obtaining the necessary information from home care patients, such as physical conditions and living environment. Students attended one out of four 1-day practicum sessions. Approximately 65 students attended each practicum and role-played in small groups of 7–8. Each group repeatedly role-played 2 cases (Table 1), with 3 to 4 students role-playing case 1, and the rest role-playing case 2.

First, the students reviewed pharmaceutical management and consultation plans as preparatory work for the role-playing, such as background confirmation for 2 patients and prescription analysis. Subsequently, they role-played checking physical conditions, including observing the patient’s general condition (complexion and limbs); confirming living conditions, such as excretion, eating, and sleeping habits; and confirming medication adherence through a medication calendar and by interview. Every student played all roles—as a pharmacist, a patient, and an observer; however, simulated patients participated in each group several times. The faculty members controlled the role-play in each group and gave feedback on the students’ communication skills, attitudes, and interview skills using an evaluation sheet. As observers, the students also checked the evaluation sheet. The students evaluated themselves using a rubric after completing the practicum, and the faculty members confirmed their responses. Moreover, the students prepared a portfolio with their self-evaluation scores and submitted it online.

### 2.3. Survey Items

#### 2.3.1. Questionnaire Survey

After completing the practicum, the students took part in a questionnaire survey that consisted of the following questions:

1. Were you able to imagine the role of pharmacists in home medical care before the practicum?
2. What are the pharmacists’ responsibilities in home medical care that you could think of before the practicum? (Multiple choices from 11 options)
3. Did the practicum improve your understanding of pharmacists’ role in home medical care?
4. Was alternating roles in role-playing helpful as a method of practical training?
5. What are the points for further learning about home patient services? (Multiple choices with 5 options)
6. After completing the practicum, do you think you can provide home-care patients with the appropriate services during practical training in pharmacies?

#### 2.3.2. Self-Evaluation Using a Rubric

After completing the practicum, the students also evaluated their own learning achievements using a 4-point rubric (Table 2). Rubrics have been described as a means to evaluate a student’s performance [14]. A rubric is a scale for evaluating learning achievements based on performance-related criteria and changes in quality. Apart from the impartiality of evaluation it offers, rubric-based learning is characterized by the instructor’s explicit presentation of outcomes to learners, which helps learners establish clear learning goals. The faculty members in charge of the practicum created the rubric in conformity with the practical training in a community pharmacy. All responses were analyzed to determine their mean ± standard deviation scores. A rubric with a target score of 3 or better was used for students’ self-evaluation of their achievements.

#### 2.3.3. Portfolio Analysis

A portfolio is a widely used documentation of student data and experiences in practicums. After the practicum, the students were requested to submit a portfolio in response to the following items:

1. What was and was not achieved through the practicum;
2. What was noticed through the practicum; and
3. Feedback for the practicum.

The portfolio design used in this study conforms to the items that the students described in their diary as recorded during their practical training in both hospital and community pharmacies. It was created by the faculty members associated with the pre-practical training. The students’ responses to item 2 were qualitatively analyzed using the grounded theory approach (GTA) [15]. The GTA is a research method that creates concepts based on data, identifies relationships between concepts, and generates a theory. We reviewed all data described in the portfolios, segmented them based on semantic contents, extracted the properties and dimensions from the segments, and determined labels for each accordingly. The relationships between analogous categories were schematized. A part of the analysis process undertaken is shown in Table 3.

## 3. Results

### 3.1. Participants’ Demographic Characteristics

The fourth-year students who participated in this study had completed classes on dispensing pharmacy and pharmaceutical healthcare sciences in community pharmacy practice up to their third year of study. They were taking classes on pharmacotherapy and practical medicine at the time of the evaluation.

### 3.2. Questionnaire Survey

**Figure 1:**
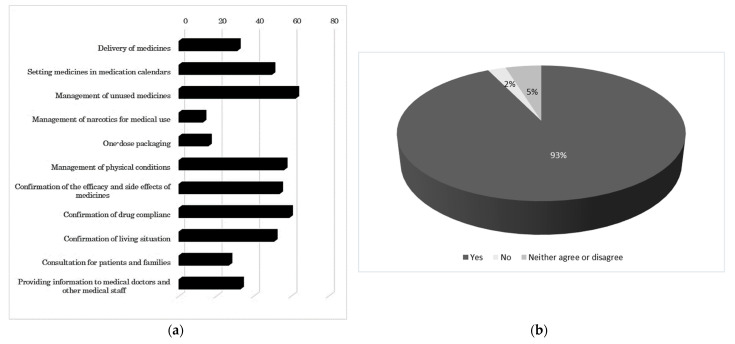
The result of the questionnaire survey: (**a**) Pharmacist duties in home medical care imagined by students before the practicum; (**b**) Effectiveness of role-playing in alternating roles. The students did not understand all the pharmacist’s roles before the practicum, especially managing narcotics for medical use and dispensing drugs in one-dose packages. Finally, 83% of the students answered that the method of alternate role-plaining was helpful.

The results of the questionnaire survey showed that 84% of the students had a general idea about the role of pharmacists in home medical care but did not have an understanding of all the tasks involved before the practicum. The students’ awareness about managing narcotics for medical use, dispensing drugs in one-dose packages, as well as providing consultation to patients and their families was particularly low (Figure 1a). After the practicum, 45% and 53% of the students reported having a full understanding and partial understanding, respectively, of pharmacists’ role in home medical care. The method of alternate role-playing was considered helpful by 93% of the respondents (Figure 1b). As points for further learning about home patient services, 36.1%, 34.4%, and 29.5% of the students selected how to complete the consultation records, how to perform physical assessments to understand a patient’s physical conditions, and what services to provide at the first visit, respectively. After completing the practicum, 60% of the students thought that they would be able to provide home care patients with the appropriate services during practical training in pharmacies, whereas 2% did not think so.

### 3.3. Self-Evaluation Using a Rubric

The students’ mean ± standard deviation rubric score was 3.0 ± 0.4, indicating that they thought they could use interviewing procedures appropriately according to not only the physical conditions of home care patients but also other factors, such as the patients’ living environment.

### 3.4. Portfolio Analysis

**Figure 2:**
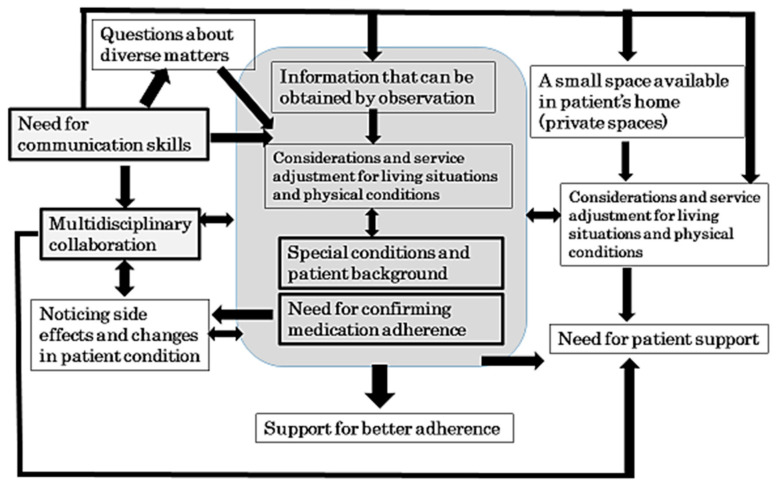
A concept map of the categories of superordinate concepts that the students noticed during the practicum on home patient service. They classified monitoring drug use, support for improving medication adherence, and observation to identify side effects early as major service categories in home medical care.

Table 4 shows the categories (as superordinate concepts) that were obtained from the analysis of the students’ responses to item 2 (“What were noticed through the practicum”) in their portfolios and the major labels constituting them. Patient conditions and backgrounds that are characteristically found in home medical care, such as advanced age, dementia, and chronic illness, as well as the services requiring consideration of an individual patient’s living settings and physical conditions, were described. For example, specific considerations are required for patients using a wheelchair. The students noted that home medical care for such patients included providing them with answers to a variety of questions based on information that they gathered from observation as well as factoring in the space restriction. As services to be provided by pharmacists, monitoring drug use through a medication calendar, support for improving adherence, as well as observation during regular visits to detect side effects and changes in patient conditions early were classified as major categories. In addition, multidisciplinary collaboration and information sharing with other healthcare and welfare personnel as well as communication skills for interactions with patients and other healthcare professionals, were listed. For communication with patients, in particular, the students stated the need for active listening, empathy, nonverbal communication, as well as creative ways and considerations to build a sense of trust, security, and hope in patients. Figure 2 shows a concept map of all the categories of superordinate concepts identified in this study.

## 4. Discussion

Pharmacists have diverse responsibilities in home medical care, of which patient-pharmacist communication is a crucial element. However, home medical care is a relatively new endeavor for community pharmacists, with students being required to receive pre-practical training before undergoing clinical training. In pharmaceutical education, role-playing through simulation is a useful learning method that allows students to acquire a broad range of knowledge, skills, and attitudes [16,17].

The questionnaire survey showed the usefulness of role-playing in the home patient services practicum and the differences between the students’ understanding of home medical care before and after the practicum. Ninety-three percent of the students who completed the practicum indicated that alternate role-playing was helpful and gave them the opportunity to observe simulated patient services by pharmacists objectively. Although the students did not repeat simulations in multiple scenarios due to the large number of participants, they felt that observing their peers and sharing in the feedback process were valuable.

The students had preconceived notions about the various responsibilities of pharmacists in home medical care, but they did not have a very good understanding of certain responsibilities, such as managing narcotics for medical use, dispensing drugs in one-dose packages, as well as providing consultation to patients and their families. After completing the home patient services practicum, 98% of the students had a better understanding of these responsibilities; however, only 60% considered themselves prepared enough to confidently provide home care to patients in their practical training. After this practicum, the students would have to undertake another practicum on services and medication instruction for patients/visitors at pharmacies (advanced), which covers at-home patient services, through which they are expected to further practice services for home care patients before starting their practical training.

The questionnaire survey administered to the students included closed, open-ended, and multiple-choice questions, with some themes overlapping with the portfolio items. We developed the questions to ascertain what the students learned from various aspects of the practicum. In addition, we quantitatively analyzed the students’ portfolios on only one theme.

Some studies have reported that the rubric assessment was useful in pharmacy education and especially in assessing student pharmacists’ communication skills [18,19]. Adrian reported that students and faculty members critiqued pharmacists’ role-playing in patient care scenarios, and grading was performed using the rubric [20]. Students demonstrated improvement in oral skills based on scores after the practicum. In this study, 90% of the students obtained the target score. We consider the rubric assessment to have been effective in facilitating self-assessment and setting goals in this practicum.

Typically, the feelings, consciousness, and values of individuals and groups are difficult to measure in a quantitative and objective manner. Qualitative research is a method of analyzing language data, such as interviews and observations, to establish generalities and universalities using independent interpretations in identifying and theorizing intrinsic meanings. Qualitative research methods incorporated in educational research allow for the evaluation of the quality of learning, for example, how the learners learned and what the learners understood. In this study, a qualitative analysis was performed on the portfolio item “What were noticed through the practicum” using the GTA, as proposed by Strauss [21]. The GTA uses formalized analytical methods and is characterized by its ability to exclude subjectivity from constructed theories as completely as possible [21]. It generally utilizes data from interviews as well as observations of videos and records, among others. As we focused on the portfolio items described in Section 2.3.3, the students’ responses tended to be superficial, indicating that their portfolios did not fully represent their internal thoughts. Studies with randomly selected students using interviewing techniques and other methods are thus warranted.

The analysis in this study revealed the following characteristics and necessary considerations in home medical care that were observed by the students through the practicum.

1. Providing services at patients’ homes, which are a private space, is different from doing so in a hospital or a pharmacy in terms of proximity between the pharmacist and the patient.
2. The unique characteristics of home medical care include the specific diseases/conditions and background of the patient, for example, stroke survivors suffering from sequelae, patients with chronic respiratory disease undergoing home oxygen therapy, and patients with dementia or terminal cancer.
3. Considerations were necessary to adjust services based on patient information from observations and interviews, such as patients’ living conditions, physical conditions, and background. In addition, medication errors, including missed doses, often pose problems in home medical care because the patients themselves or nonprofessional caregivers such as their family members manage their medication [7].
4. The students perceived the need for pharmacists’ support to monitor unused and used drugs and to improve patients’ medication adherence through the use of medication calendars and patient interviews.
5. Because medical staff is not always accessible in home medical care, pharmacists should not only carefully observe patients during visits to check for any side effects or changes in medical condition but also share the information with other healthcare professionals, including medical doctors, nurses, and caregivers.
6. Many students reported that they noticed the importance of communication skills in obtaining necessary information from patients and in collaborating with various healthcare professionals.

In 1996, Schneider and Barber [22] reported on the provision of a domiciliary service by community pharmacists in the United States. The pharmacists who visited patients’ homes found medication nonadherence, medication hoarding, and adverse drug reactions upon checking the medications in the patients’ possession and based on their medication records. In spite of the emerging need for pharmacist-led home visits, these are currently not standard practice in the community pharmacy setting [10]. Furthermore, Ensing et al. [23] reported that pharmacists’ communication skills were key to improving the pharmacist-patient interaction during a post-discharge home visit.

In this study, we evaluated the usefulness of a home patient services practicum based on a 6-question survey, a rubric assessment, and a portfolio assessment. We stipulated that the use of multiple instruments would lead to synergistic effects. The questionnaire survey confirmed the students’ level of understanding of the role of pharmacists in home medical care before and after the practicum as well as their readiness for practical training. The rubric assessment quantitatively measured the students’ learning achievement. Finally, the portfolio assessment qualitatively analyzed the students’ perceptions about the practicum.

An important strength of this study is its evaluation of the usefulness of a new practicum. The simulated role-playing allowed the students to experience being a community pharmacist in home medical care.

A limitation of this study is that it did not thoroughly evaluate the students’ skills and attitudes. Although the students’ points of view were collected, the faculty members’ objective evaluations were not analyzed. Another limitation of this study is its design. The primary objective of this study was to assess the knowledge, skills, and various realizations that the students gained during the practicum, but changes in these parameters from before and after the intervention were not examined; the OSCE results and the outcomes of the practical training were also not considered. Kimberlin reported that various assessment forms for communication skills were used in the US College of pharmacy [24]. Additionally, faculty concerns were a lack of continuity and congruence of assessment across the curriculum. In addition, a qualitative analysis using the GTA was performed only on one portfolio item (“What were noticed through the practicum”), thus rendering further multifaceted analysis of other items desirable.

The results of this study are useful in order to create a new home patient services practicum in pharmaceutical education. Role-playing as a practicum method should be introduced to obtain communication skills with patients and manage the pharmaceutical service at patients’ homes.

## 5. Conclusions

The practicum in home patient services as a pre-learning opportunity for practical training in pharmacies would allow students to (i) understand patients’ characteristics and backgrounds and thereby adjust their services accordingly, (ii) check for side effects and changes in medical conditions during visits, as well as (iii) share information with other healthcare professionals. This study also highlights the value of proper communication with patients and other healthcare professionals in improving the students’ readiness for practical training.

## Figures and Tables

**Table 1:** Details of Cases role-played by students. The students role-played one of the two cases. One was the patient with post-cerebral infarction sequelae, and the other was the patient with chronic obstructive pulmonary disease.

	Case 1	Case 2
Patient	A 70-year-old woman with post-cerebral infarction sequelae	A 68-year-old man with chronic obstructive pulmonary disease
Past medical history	Hypertension	No appreciable history
Characteristic patient background	Paralysis of the left lower limb	Shortness of breath during exertion, chronic cough/sputum Home oxygen therapy when necessary Use of an inhaled drug
Points for patient services	Confirmation of living situation Considerations about paralysis Confirmation of adherence	Considerations about living situation Confirmation of the used amount of the inhaled drug

**Table 2:** Rubric-based self-evaluation by students. The students evaluated their learning achievement using a rubric after completing the practicum.

Understanding Information on Home-Care Patients	4	3	2	1
Capable of using appropriate procedures to obtain necessary information from home-care patients, such as physical conditions and living environment	Capable of understanding patient condition through evaluation of necessary information about home-care patients, such as physical conditions and living environment, collected using appropriate procedures	Capable of using appropriate procedures of information collection selected with consideration for not only physical conditions but also living environment	Capable of exhaustively obtaining minimum information that should be collected	Capable of identifying pieces of information that should be collected based on existing patient information

**Table 3:** An example process of analysis using GTA. All data were described in the portfolios and were segmented. The properties and dimensions from the segments were extracted, and the labels were determined, then the categories were grouped.

Segment	Data	Property	Dimension	Label Name	Category
269	If a pharmacist is aware of visiting a patient’s home, he/she should emphasize the speed, loudness, and ease of hearing of his/her speech to the patient. Patients trust a pharmacist who speaks as slowly as possible, uses easy-to-understand words, and has good facial expressions while interacting, and a pharmacist who interacts more closely with the patient can obtain more information about the patient.	Setting Emphasis Patient’s feelings	Patient’s home Loudness, speed, and ease of hearing of speaking Speak as slowly as possible Speak using easy-to-understand words Have good facial expressions while interacting Trust pharmacist, interact closely	Ways of speaking to get patients to trust	Improvements in communication
582	In home medical care, it is important to understand physical conditions and living situations of patients. The basic living situations can have a significant impact on treatment. For example, diet is a major key factor for patients with diabetes.	Things to understand Basic living environment Patients with diabetes	Patients’ physical conditions and living situations Relevant to treatment Diet is a key	Understanding living situations and treatment	Considerations and responses to living situations and physical conditions
237	When a patient’s drug compliance is poor, there can be reasons, e.g., drugs are difficult to take, and the patient does not know how to use the drugs; so I thought it was important to identify the cause through communication with the patient and take measures to correct it.	Situations Possible reasons Important elements	Noncompliance Drugs are difficult to take; methods of use are unknown Identify the cause through communication and take measures to correct it	Search for the cause of noncompliance	Medication support to improve adherence
82	I felt that communication with other medical staff is going to be more important because sharing information obtained during the visit with the medical staff involved in home medical care of the patient is important to support the patient.	Interactions with other medical staff	Need for sharing patient information and communication	Interactions with other medical staff	Multidisciplinary collaboration

**Table 4:** Major categories and labels. The labels and categories were obtained from the analyses of the students’ response to “What were noticed through the practicum”.

Major Labels	Categories
Providing services to patients with dementia	Special conditions and patient background
Restricted life due to chronic illness or advanced age	
Patients feeling inconvenience in life	
Complexity of patient background	
Noticing small changes in patients	Information that can be obtained by observation
Gathering information through questions and observations	
Observation of facial expressions	
Observation of general conditions during home visits	
Checking living environment and adjusting services to patients	Considerations and service adjustment for living situations and physical conditions
Eating, medication schedule, and sleep duration	
Interviewing patients about ADL-related items	
Consideration for patients’ feelings and physical conditions	Considerations tailored to individual patients
Consideration for patients using wheelchairs	
Serious symptoms and considerations for patients	
Word choice for checking elimination	
Consideration for personal information and right to autonomous decision making	
Considerations in patients’ private spaces	A small space available in patient’s home
Interviews taking advantage of personal space	
Manners in daily life settings	
Questions that should be asked and delving deeper into questions	Questions about diverse matters
Effectiveness of medication calendar for elderly patients	Need for confirming medication adherence
Checking inhaled drug usage	
Specific methods of confirming used and unused drugs	
Checking and addressing cause of unused drugs	
Regular visits to maintain medication adherence	Support for better adherence
Search for causes of noncompliance	
Improvements in ease of taking drugs	
Advice and plans for patients who cannot self-manage medicines	
Finding side effects through regular visits	Noticing side effects and changes in patient conditions
Early detection through observation and monitoring and taking corrective actions	
Rapid detection of abnormalities in home-care patients in the absence of healthcare professionals	
Interviewing patients about their living situations jointly with other healthcare professionals	Multidisciplinary collaboration
Sharing limited information obtained during visits	
Collaboration with healthcare and welfare personnel	
Bridge between patients and medical care	
Considerations when talking to patients	Need for communication skills
Active listening about patients’ feelings	
Ways of speaking to gain patients to trust	
Nonverbal communication and active listening	
Attitude showing respect for patients	
Atmosphere that facilitates conversation	
Communication to eliminate anxiety	
Giving positive words to patients whose conditions cannot be cured	
Significance of praising patients	

## Data Availability

Data available on request due to restrictions, e.g., privacy or ethical. The data presented in this study are available on request from the corresponding author. The data are not publicly available.
